# Short‐Term Effect of Air Pollutants on Sudden Unexpected Death in Infancy: A Case‐Crossover Study Based on the French National Registry

**DOI:** 10.1111/ppe.70196

**Published:** 2026-09-07

**Authors:** Emmanuel Bourdet, Noémie Letellier, Tarik Benmarhnia, Johanna Lepeule, Bénédicte Jacquemin, Karine Levieux, Guillaume Barbalat, Itai Kloog, Rémi Béranger, David Boels

**Affiliations:** ^1^ Univ Rennes, Inserm, Irset (Research Institute for Environmental & Occupational Health) UMR 1085, Rennes France; ^2^ Service de Santé Publique et Environnementale, CHU Nantes, Nantes Université Nantes France; ^3^ Université Paris‐Cité, INSERM, Optimisation thérapeutique en neuropharmacologie OTEN U1144 Paris France; ^4^ Scripps Institution of Oceanography UC San Diego USA; ^5^ Team of Environmental Epidemiology Applied to Development and Respiratory Health, Institut pour l'Avancée des Biosciences (IAB), INSERM U1209, CNRS UMR 5309, Université Grenoble Alpes Grenoble France; ^6^ Pediatric Emergency Care Unit, Nantes University Hospital Nantes France; ^7^ Centre Ressource de Réhabilitation Psychosociale et de Remédiation Cognitive, Hôpital Le Vinatier, Pôle Centre Rive Gauche, UMR 5229 CNRS & Université Claude Bernard Lyon 1 France; ^8^ Department of Environmental Medicine Icahn School of Medicine at Mount Sinai New York New York USA; ^9^ Univ Rennes, CHU Rennes, Inserm, Irset (Research Institute for Environmental & Occupational Health) UMR 1085 Rennes France

**Keywords:** air pollution, environmental exposure, infant mortality, sudden unexpected death in infancy

## Abstract

**Background:**

Sudden unexpected death in infancy (SUDI) remains poorly understood, and the potential contribution of air pollution exposure is largely unexplored. Given its known impact on infant health, air pollution may influence the risk of SUDI, particularly through short‐term effects.

**Objectives:**

We aimed to evaluate the short‐term effect of exposure to ambient air pollutants on SUDI in France.

**Methods:**

Using data from the French national SUDI registry (2015–2022) and modelled air pollution data, we assigned daily concentrations of particulate matter (PM_10_ and PM_2.5_, μg/m^3^), nitrogen dioxide (NO_2_, μg/m^3^) and ozone (O_3_, μg/m^3^) at each infant's residence. We conducted a time‐stratified case‐crossover analysis comparing single‐ and multi‐pollutant exposure on the day of death with matched control days, considering lagged effects up to 6 days. Odds ratios (ORs) and 95% confidence intervals (CIs) were estimated using conditional logistic regression, adjusted for public holiday, influenza‐like syndrome and daily mean temperature. We assessed the potential effect of several effect modifiers, such as biological sex, age at death, season, urban or rural residence, social deprivation and sleeping position at discovery.

**Results:**

A total of 1078 SUDI cases were included. Exposure to PM was consistently associated with higher odds of SUDI, with a lag effect of 3 to 5 days before death and during the last week of life. NO_2_ exposure presented a trend of association. Higher effects were observed for PM and NO_2_ with cumulative exposure, especially for PM and consistent with the multi‐pollutant model. We observed seasonal heterogeneity, with higher ORs in the spring for PM and O_3_.

**Conclusions:**

Our findings support a plausible short‐term trigger effect of air pollution on SUDI. These results highlight the need to integrate environmental determinants, particularly for NO_2_ and PM, into SUDI prevention strategies.

## 1 Background

Sudden unexpected death in infancy (SUDI) is one of the leading causes of mortality between 1 month and 1 year [1, 2]. SUDI is defined as the unexpected death of an infant before 1 year of age [3]. Although post‐mortem investigations can identify various factors contributing to the death, some cases remain completely unexplained and are classified as sudden infant death syndrome (SIDS). The majority of studies focus on SIDS, which has made it possible to identify the predominant risk factors, including smoking exposure, preterm birth, bed sharing, soft bedding and the prone sleep position [1]. SUDI cases not classified as SIDS are poorly studied. Although postmortem investigations identify risk factors and vulnerabilities, they often fail to present sufficient evidence of the cause of death.

Two recent meta‐analyses presenting results on SIDS and ambient air pollution highlighted an increased risk of SIDS with long‐term exposure to PM_10_ and short‐term exposure to NO_2_, PM_10_, SO_2_, CO and O_3_ [4, 5]. Although air pollution represents a significant burden for infant health, SUDI findings remain limited, inconsistent for the different pollutants, and focus on SIDS. This deficiency is particularly alarming given infants' vulnerability and increasing air pollution due to climate change [6]. In addition, air pollution presents a plausible potential biological effect on SUDI. Short‐term exposure to air pollutants may disrupt autonomic nervous system regulation, a mechanism frequently proposed in the pathophysiology of SIDS [4]. Air pollution can induce systemic inflammation, oxidative stress and autonomic imbalance, which may impair cardiorespiratory control in vulnerable infants [7]. Within the framework of the main mechanistic hypothesis for SIDS, air pollution may act as a triggering factor, interacting with an underlying vulnerability during a critical development period, and initiating an inflammatory and neurotoxic cascade [8, 9].

In addition to the plausible effect of air pollution on SUDI, effect modifiers could interfere with associations. First, known risk factors for SUDI, including biological sex or age at death, could amplify the effect of air pollution by increasing vulnerability [10]. Existing studies on air pollution have reported a higher risk of SIDS among female infants and age at death above 3 months [11]. The main SUDI risk factor, the prone or lateral sleep position, can lead to hypercapnia, inflammation, and dysregulation of the autonomic nervous system [9, 10]. As air pollution can present similar effects, including neuroinflammation, oxidative stress, or apnea, the cumulative effect of exposure and position could increase the risk of SUDI [12, 13]. Factors such as urbanicity or social deprivation could represent different exposures and susceptibility, and identify vulnerable subpopulations and health burdens in a context of environmental inequity [14, 15, 16]. Finally, seasons can vary the effects of air pollution by changing its composition, especially PM composition, through temperature effects or additional pollutants (e.g., pollen and pesticides) [17, 18].

In France between 2005 and 2015, the rate of SUDI was 50.3 per 100,000 live births, one of the highest in Western Europe [2]. Although the majority of studies on air pollution and SUDI in the international literature have focused on SIDS, France created a registry in 2015 that includes all national SUDI cases [19]. The lack of knowledge on potential air pollution effects on SUDI limits the ability to develop prevention strategies. In this study, we aimed to analyse the short‐term effect of air pollution on SUDI in France. In addition, we assessed the role of several potential effect modifiers: biological sex, age at death, season, urban or rural residence, social deprivation, and sleep position at discovery.

## 2 Methods

We performed a time‐stratified case‐crossover study to analyse the short‐term effect of air pollution exposure on SUDI.

### 2.1 Study Data and Outcome

SUDI data were provided by the French SUDI registry (*Observatoire de la Mort Inattendue du Nourrisson*). The registry has been collecting data on SUDI cases in France since 2015. The data include administrative information such as age, biological sex, date of birth, date of death, place of residence and socioeconomic data. Medical information includes the health histories of the infant and parents, autopsy reports, diagnoses, risk factors (e.g., sleep position), and classification (especially to identify SIDS cases) [20]. All data from the registry are collected through interviews with parents, investigations, and the referring doctor's conclusions.

We included all SUDI cases available in the registry with a residential address between 2015 and 2022 (*n* = 1273). Precision of residence varied from GPS coordinates (69.9%) to street (15.2%) and to town/village (14.9%). We excluded cases older than 12 months (*n* = 130) in order to respect the international definition of SUDI, as well as cases reported in a different and not adjacent *département* (French equivalent of a county) to the place of residence (*n* = 32). A multidisciplinary group of experts screened all autopsy reports from the French registry and excluded SUDI cases with clearly identified non‐environmental causes, such as traumatic deaths and intoxication (*n* = 41). Screeners had both clinical and research expertise, as EB, DB, RB and KL are a nurse anaesthetist/epidemiology PhD student, a pharmacist/toxicologist, a midwife/epidemiologist, and a paediatrician/coroner, respectively. Each expert classified the reports separately. In the event of a disagreement, a discussion was held until a consensus was reached (Figure S1).

### 2.2 Exposure

We considered the following exposures in our analysis: particulate matter with an aerodynamic diameter ≤ 10 μm (PM_10_) and ≤ 2.5 μm (PM_2.5_), nitrogen dioxide (NO_2_), and ozone (O_3_). Daily NO_2_ (μg/m^3^) estimates in France were obtained from a validated multi‐stage ensemble model with an overall 1 × 1 km^2^ resolution, and a 200 × 200 m^2^ resolution in urban areas [21]. PM (μg/m^3^) and O_3_ (μg/m^3^) were modelled by INERIS (*Institut national de l'environnement industriel et des risques*) on a 2 × 2 km^2^ grid, using both a chemistry‐transport model and kriging methods [22]. Daily temperature was estimated using a model operated by the French national weather service (*Météo France*) with an 8 × 8 km^2^ spatial resolution [23]. We did not develop an air pollution level for this analysis; instead, we used existing data from various sources at various resolutions.

We used QGIS (QGIS Desktop 3.32.3) to assign pollutant exposure levels to each SUDI residential address declared for the day of death (i.e., the value of the corresponding pixel or cell in each pollutant grid). For NO_2_ in urban areas, air pollution exposure was assigned using the 200 × 200 m^2^ grid when the SUDI residence was located within 150 m of the grid centroid; otherwise, exposure was assigned using the 1 × 1 km^2^ data. For every SUDI case, we matched pollutant concentrations estimated for each day using the time‐stratified methodology, including lags. We obtained the daily concentration for case and control days for each SUDI case.

### 2.3 Effect Modifiers

Age (0–2 or ≥ 3 months), sleep position (prone or side/other positions), biological sex (female or male), and residence (urban or rural) were defined as binary variables. Age categorisation was defined based on previous similar studies [11, 24]. As the side sleep position increases the risk of SIDS [10], we included it in the analysis together with the prone position. Urban or rural residence was identified using the classification of the French Institute for Statistics and Economic Studies (*Institut national de la statistique et des études économiques*), in which rural refers to areas with fewer than 2000 inhabitants (https://www.insee.fr/fr/information/4802589). Social deprivation was defined by the quintiles of the French deprivation index (Fdep), with the first quintile representing the wealthiest areas and the fifth the most deprived. Fdep is an ecological index generated at the neighbourhood level from several variables, including the unemployment rate, median household income, and the proportions of non‐graduates and manual workers in the area [25].

### 2.4 Statistical Analysis

We assessed correlations among pollutants using Spearman's correlation and presented the results as a heatmap. We performed the case‐crossover analysis to assess the association between air pollution and SUDI. The case‐crossover design compares the day of the event with a set of comparable control days to estimate the short‐term effect of air pollution. This design implicitly addresses confounding by time‐invariant factors (e.g., sex and age), because the case and control days involve the same infant, thereby adjusting for these characteristics by design. We conducted a time‐stratified design to limit seasonal, long‐term and overlap biases [26, 27, 28]. In this design, control days are the same day of the week, within the same month and year as cases [28, 29]. We performed Akaike information criterion (AIC) of nested models to assess the potential non‐linearity of the association. We then tested knots up to six using natural cubic splines. As the model without knots presented the lowest AIC for all pollutants, we were able to exclude a non‐linear association and we calculated the odds ratio (OR) and 95% confidence interval (CI) from conditional logistic regression for each interquartile range (IQR) increase in pollutant concentration.

All models were adjusted for daily mean temperature, public holidays and influenza‐like syndromes, as these time‐varying factors could bias the potential exposure‐outcome relationship. Public holidays could modify behaviour and exposure; influenza‐like syndromes, mortality risk and vulnerability; and temperature could affect both health effects and air pollution. Data on influenza‐like syndromes were obtained from the French open‐access data service *Sentinelles* (https://www.sentiweb.fr/) and included standardised incidence rates per 100,000 inhabitants for each of the 22 regions of France. It is calculated based on the number of visits for fever and respiratory symptoms.

Lag effects were included up to 6 days before death. Lag structures were selected based on previous literature suggesting both immediate and delayed physiological responses to air pollution exposure in infants. Each day lag from 0 to 6 was analysed in a separate model. We also included the average exposure over several days (lag 0–1: day of death and the day before; lag 0–2: day of death and the 2 days before; up to lag 0–6) to assess the cumulative exposure effect.

We then performed stratified analysis to assess several potential effect modifiers, including biological sex, age at death, social deprivation, urban or rural residence, position at discovery and season. Factors were defined based on their potential effect and the availability of data in the registry. We assessed heterogeneity across strata using Cochran's *Q* test [30, 31]. For each stratum, we used the time‐period lags 0–1, 3 and 0–6 to assess the acute, medium and cumulative effects.

To estimate the mixture effect of pollutants, we used a multi‐pollutant model based on quantile g‐computation [32, 33]. We then incorporated the coarse part of PM_10_ (PM_10_–PM_2.5_), PM_2.5_, NO_2_ and O_3_ into the model and assessed the effect of a 1‐quartile increase in each pollutant.

All analyses were conducted with RStudio software (version 4.1). An extraction of the R code used is provided in the supplemental material.

#### 2.4.1 Sensitivity Analysis

Because the SUDI residential addresses had varying precision and introduced potential classification bias, we focused on a single sensitivity analysis using data with precise GPS coordinates (*n* = 753). In addition, we performed analyses without adjustment (*n* = 1078), and analyses without any exclusion (*n* = 1273), as our exclusion criteria could introduce a selection bias, particularly through screening and classification methods.

To compare with our cumulative effect method, which averages across different exposure lags, we fitted a distributed lag model (DLM) to assess the overall cumulative effect and the lag effect. We then used the *dlnm* package (https://cran.r‐project.org/web/packages/dlnm/dlnm.pdf) in R and defined a cross‐basis with a linear dose–response relationship between SUDI and air pollution. We assessed the number of knots in lag‐response natural splines using AIC and readability. We presented results for one (lag 3), two (lags 1 and 5) and three (lags 1, 3 and 5) knots.

#### 2.4.2 Missing Data

Some potential effect modifiers data were missing: biological sex (0.2%), sleep position at discovery (12.2%) and Fdep (0.1%). We conducted multiple imputations for stratified analyses: we then performed 50 imputations based on existing data distribution and pooled the estimates.

### 2.5 Ethics Approval

We obtained ethical approval from the scientific committee of the French SUDI registry, the French data protection authority (CNIL) and the French ethics and scientific committee for health research (CESREES) (CNIL, approval no. 924124).

## 3 Results

We included 1078 SUDI cases in the main analysis (Figure S1). Most infants were male (57.7%), and nearly half of the deaths occurred between zero and 2 months of life (47.2%). The majority of infants lived in urban areas (81.9%). At the time of discovery, 33.4% were found in the prone position and 11% on their side. Regarding social deprivation, 25.1% resided in the most deprived Fdep quintile (Table 1). Table 2 presents the distribution of air pollutants, with mean concentrations at 16.6 μg/m^3^, 10.2 μg/m^3^, 17.6 μg/m^3^ and 52.6 μg/m^3^ for PM_10_, PM_2.5_, NO_2_ and O_3_, respectively. Mean PM and NO_2_ concentrations were higher in winter, while maximum concentrations were observed in spring. O_3_ presented higher levels in spring and summer (Table S3).

**TABLE 1:** Description of participants' characteristics (*n* = 1078).

Characteristics	Number of cases	Percentage (%)
*Biological sex*		
Female	454	42.1
Male	622	57.7
Unknown	2	0.2
*Age at death (in months)*		
0–2	509	47.2
3–12	569	52.8
*Season of death*		
Winter	261	24.2
Spring	259	24
Summer	245	22.7
Autumn	313	29
*Urban/rural*		
Urban	883	81.9
Rural	195	18.1
*Fdep*		
Q1 (wealthiest)	166	15.4
Q2	177	16.4
Q3	226	21
Q4	237	22
Q5 (most deprived)	271	25.1
Unknown	1	0.1
*Sleep position at discovery*		
Prone	360	33.4
Side	118	11
Other	586	43.4
Unknown	132	12.2
*Public holiday*		
Day of the death (*n* = 1078)	26	2.4%
Control days (*n* = 3364)	135	4.0%

*Note:* Social deprivation was assessed using the French deprivation index (Fdep) in quintiles, with the first quintile representing the wealthiest areas and the fifth the most deprived.

**TABLE 2:** Distribution of air pollutants, temperature and influenza‐like syndromes.

	Mean (SD)	Percentile					IQR
		Minimum	25th	50th	75th	Maximum	
PM_10_ (μg/m^3^)	16.56 (8.3)	2.9	10.9	14.6	20.2	86.9	9.3
PM_2.5_ (μg/m^3^)	10.2 (6.9)	1.6	5.7	8.0	12.5	72.6	6.8
NO_2_ (μg/m^3^)	17.65 (13.7)	0.0	7.3	13.9	24.5	104.8	17.2
O_3_ (μg/m^3^)	52.62 (21.3)	0.0	38.5	53.3	67.2	134.3	28.7
Mean temperature (°C)	12.5 (6.6)	−4.8	7.7	12.0	17.3	31.6	9.6
Influenza‐like syndromes (standardised incidence per 100,000 inhabitants)	65.3 (113.7)	0.0	4.0	20.0	62.0	864.0	58.0

We observed a strong correlation between PM_10_ and PM_2.5_ (*r* = 0.89), and moderate correlations between NO_2_ and PM_2.5_ (*r* = 0.45) and between NO_2_ and PM_10_ (*r* = 0.41). Mean temperature was positively correlated with O_3_ (*r* = 0.55) and negatively, but weakly, correlated with PM_2.5_ (−0.26) (Figure S2).

Overall, positive associations were identified between SUDI and exposure to PM and NO_2_ (Figure 1 and Table S2). We observed positive associations for all lag days for PM, particularly for PM_10_ at lag 0–6, lag 0–5, lag 0–4 and lag 3. PM_2.5_ showed a pattern similar to PM_10_. Findings about NO_2_ exposure showed positive associations, with a wider confidence interval than PM. Highest NO_2_ results concerned lags 0–6, 0–5 and 0–4. O_3_ exposure did not show an association with SUDI (Figure 1 and Table S2).

**FIGURE 1:**
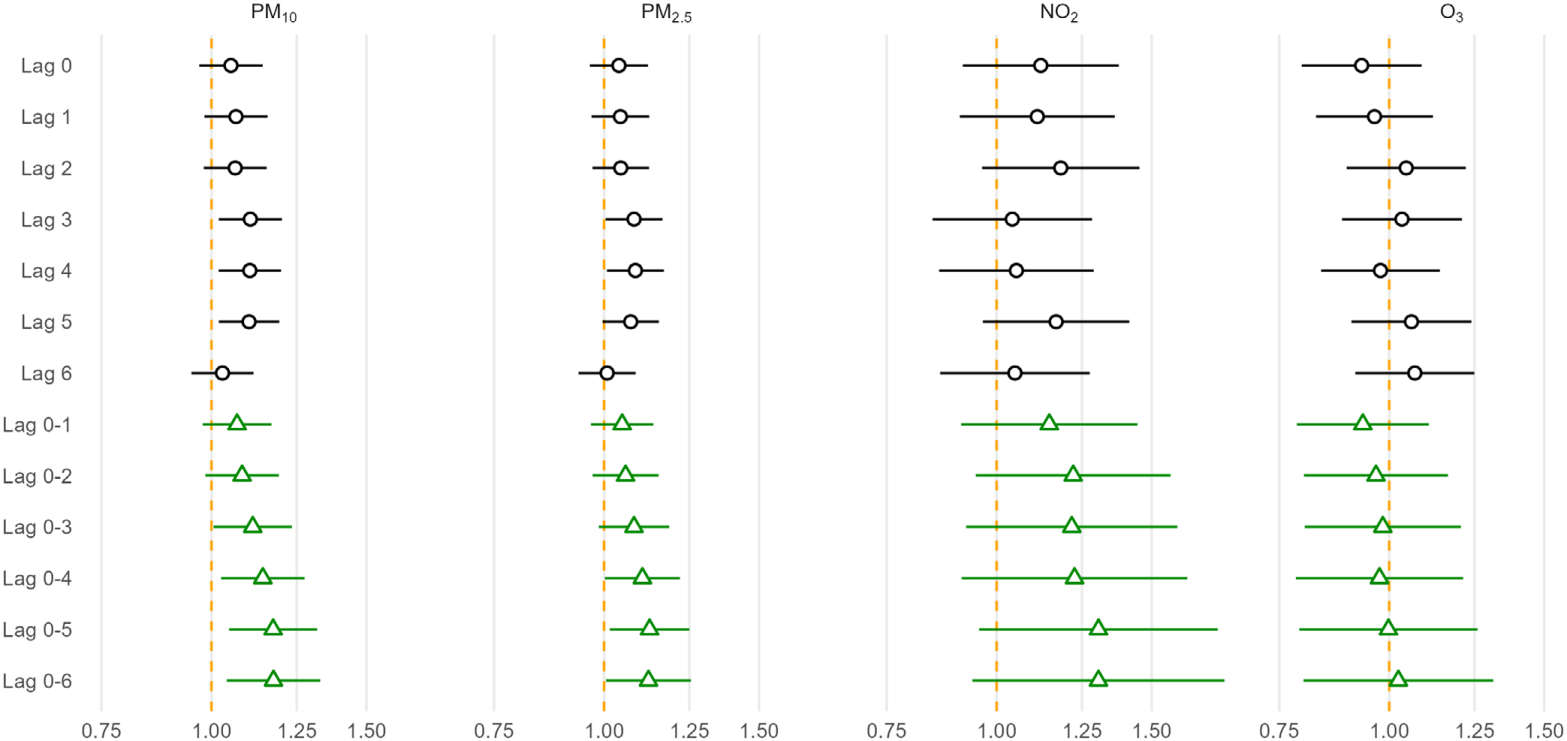
Odds ratio of sudden unexpected death in infancy (SUDI) associated with an interquartile range (IQR) increase in air pollutants, adjusted for mean temperature, influenza‐like syndromes and public holiday. Influenza‐like syndromes were defined as the incidence per 100,000 inhabitants in the administrative region of residence. The odds ratios and 95% confidence intervals in black correspond to single lags, and those in green correspond to average exposures over several days.

Regarding stratified analyses, we did not observe notable heterogeneity across strata (Table S4 and Figures S3–S6), except for social deprivation and season (Table S4). The qualitative analysis of results from social deprivation is inconsistent due to variability between the different quintiles. In brief, NO_2_ exposure showed higher odds for the second quintile, while O_3_ presented lower odds for the first and second quintile at lag 0–1 and higher odds for quintiles 3 and 4 at lag 0–6 and 3 (Figure S7). Regarding season, PM and O_3_ exposure were associated with higher odds of SUDI in spring (Figure 2).

**FIGURE 2:**
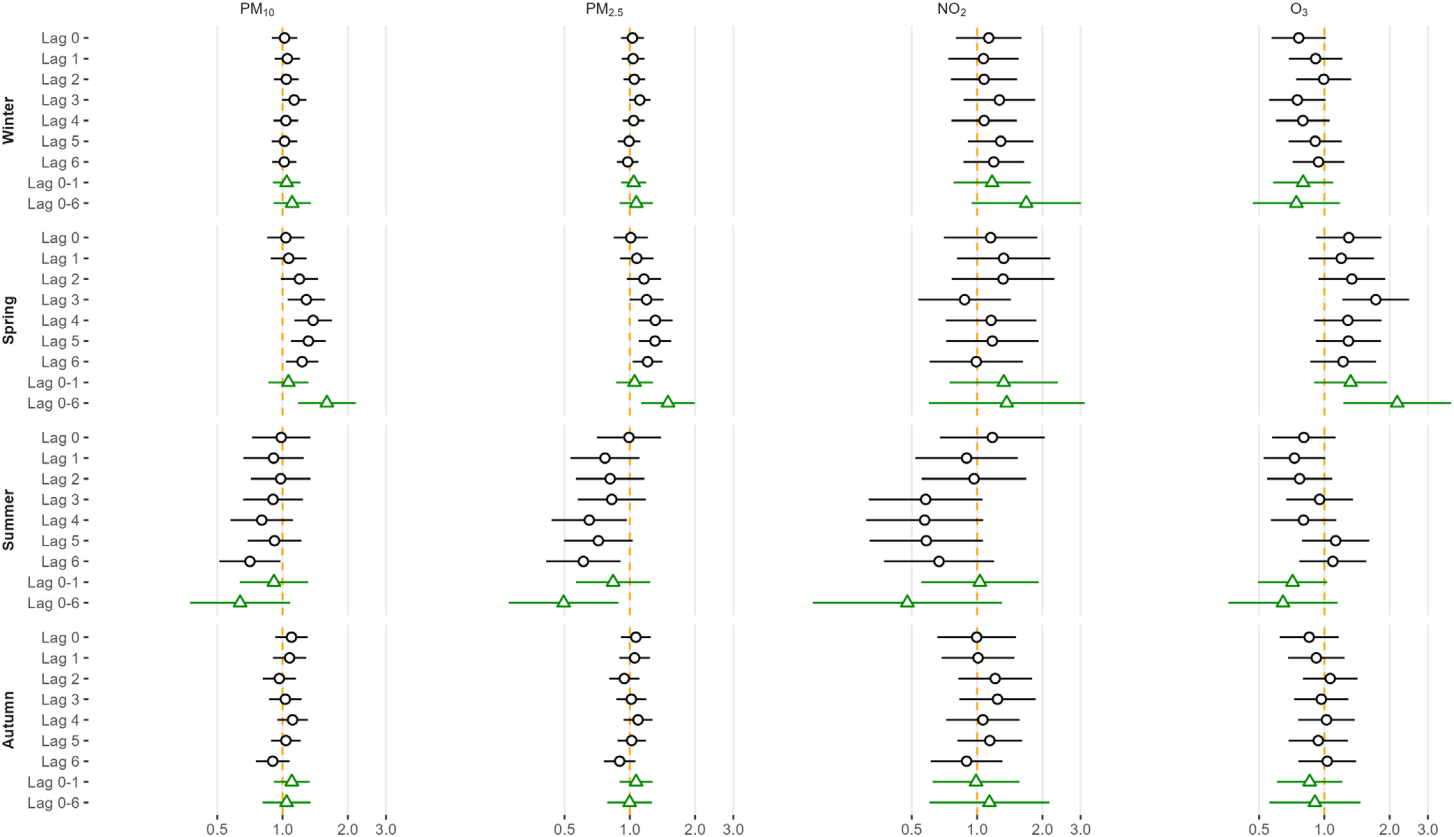
Odds ratio of sudden unexpected death in infancy (SUDI) associated with an interquartile range (IQR) increase in air pollutants, adjusted for mean temperature, influenza‐like syndromes and public holidays, stratified by season at death. Influenza‐like syndromes were defined as the incidence per 100,000 inhabitants in the administrative region of residence. The odds ratios and 95% confidence intervals in black correspond to single lags, and those in green correspond to average exposures over several days.

The multi‐pollutant analysis showed an impact of air pollution at lag 1, 3 and 6, and particularly at lag 5. Findings concerning cumulative exposure presented a trend of associations, but with a wider confidence interval than single lags (Table S1 and Figure 3). Other lags also showed positive associations, with a wide confidence interval. Weight distribution revealed a positive effect of PM_10_ and PM_2.5_ at each lag, and a more variable positive or negative contribution of NO_2_ and O_3_ (Figure S8).

**FIGURE 3:**
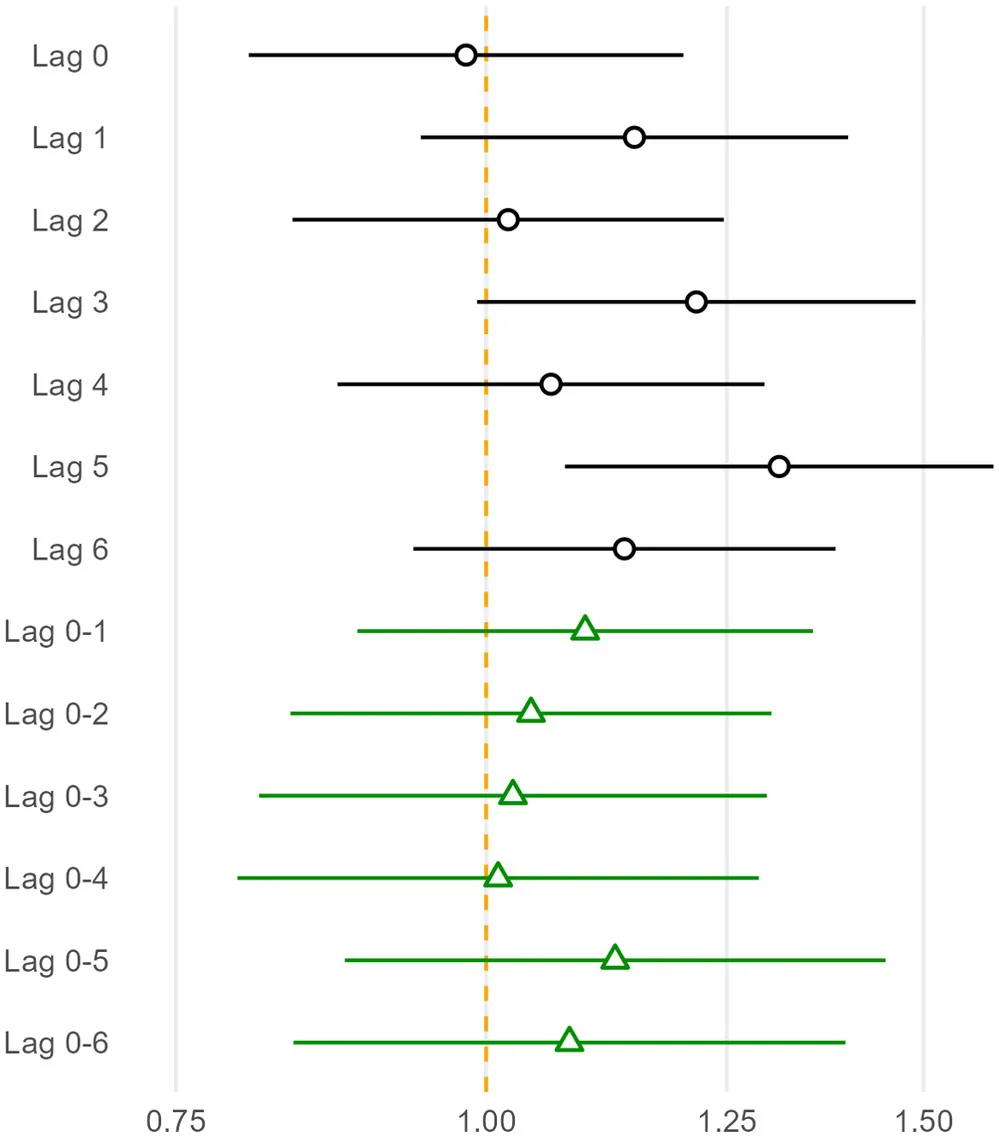
Odds ratio of sudden unexpected death in infancy (SUDI) associated with a quartile increase in mixture pollutants, adjusted for mean temperature, influenza‐like syndromes and public holiday. Influenza‐like syndromes were defined as the incidence per 100,000 inhabitants in the administrative region of residence. The odds ratios and 95% confidence intervals in black correspond to single lags, and those in green correspond to average exposures over several days. Each OR correspond to a quartile increase of all pollutants: Coarse part of PM_10_, PM_2.5_, NO_2_ and O_3_.

### 3.1 Sensitivity Analysis

When we focused on data with precise GPS coordinates, data without exclusion and crude analyses, we observed the same pattern as in the main analysis (Figures S9–S11). DLM results showed that PM had an impact on SUDI, especially at lag 4 and 5. NO_2_ effects were observed across all lags, with stronger associations at lags 5 and 6. We observed no association with O_3_. Overall, cumulative DLM estimates showed consistent results with the main analysis (Figures S12–S15).

## 4 Comment

### 4.1 Principal Findings

This study suggests that short‐term exposure to air pollution is associated with a higher risk of SUDI, especially for cumulative PM exposure and from lags 3 to 5. Multi‐pollutant analyses support the effect of PM. DLM analyses are consistent with the main analyses for all pollutants, but the model's over‐adjustment could limit precise interpretation. PM and O_3_ effects were higher in spring.

### 4.2 Strengths of the Study

We proposed a study focused on environmental exposures in SUDI, which remain largely poorly understood and understudied. This is the first study to assess the association between air pollution and SUDI in France, and is based on a single‐ and multi‐pollutant analysis. Our study population was larger than in previous similar studies, which included hundreds of SIDS cases, whereas we were able to include 1078 SUDI cases. In addition, we had access to several variables provided by the French registry, allowing us to assess effect modification. We included all SUDI cases without a clearly identified trigger cause. This approach could be applied in other research and is not limited to SIDS cases, as triggering mechanisms may be similar across different SUDI profiles.

### 4.3 Limitations of the Data

Although our population is large for a rare outcome, it represents a small volume in statistical terms. It posed difficulties for performing stratified analyses, especially when we included more than two strata, such as social deprivation. This limitation could be improved by developing a multi‐country study.

The exposure assessment presents very different modelling resolutions, from 200 m for NO_2_ in urban areas to 8 km for temperature. Using a high‐resolution model for all metrics could reduce misclassification bias and increase the ability to observe the effects of air pollution. Similarly, the precision of the residence address is crucial to improving the quality of exposure prediction and analysis. When we focused on the most precise address (i.e., GPS coordinates), we observed a stronger association, suggesting a dilution effect across different levels of precision. Of course, using only the residence to assess exposure has limitations because we assume infants spend most of their time at home, even though they may also spend substantial time in other settings. This may involve exposure misclassification bias; reducing the precision of results or diluting the potential associations. In addition, we focused on outdoor air pollution, while indoor air pollution represents an important source of exposure for the population and poses a challenge for perinatal health.

Although PM is defined by its size and contains a wide range of compounds, it remains unclear which specific role any given compound plays. Using all PM, including PM_2.5_, in a study could lead to underestimation or overestimation of the effect. In addition, to improve the causal validity of etiological studies, examining both the identified compounds and the overall mixture could help target interventions more effectively, such as preventive measures at a specific pollution source, and ultimately reduce adverse health effects [18].

### 4.4 Interpretation

Previous international studies using a comparable design observed similar patterns in the effects of air pollution on SIDS. Hwang et al. [11] in South Korea and Litchfield et al. [34] in the United Kingdom reported effects of NO_2_ and PM_10_ exposure, particularly with a lag effect from 2 to 5 days, consistent with our findings. Although this temporal pattern aligns with the theoretical plausibility of a delayed onset of systemic and neuro‐inflammatory responses following particle inhalation, the precise biological mechanisms driving this specific 2–5 days window remain to be fully elucidated in infant populations.

Although the main analysis showed an OR close to the null for O_3_, we observed potential effect modification by season, sleep position and social deprivation, suggesting the overall estimate may mask a true effect restricted to specific exposure windows or susceptible subgroups. This is biologically plausible given ozone's marked seasonal variability, as well as its known synergistic interactions with co‐exposures such as aeroallergens and ambient temperature, which are themselves seasonal. However, given the limited statistical power of subgroup analyses for a rare outcome such as SUDI, and the number of strata examined, chance findings and reduced precision of interaction estimates cannot be excluded, and these results should be interpreted as hypothesis‐generating.

The spring season showed higher odds for PM and O_3_. Regarding O_3_, increased sun exposure and time spent outside could increase both the duration and the total exposure. Particulate matter, particularly related to spring, such as pollen or pesticides, could also play a role. Regarding PM specifically, this highlights the need to assess PM composition.

Although social deprivation is known to increase both exposure and health effects of air pollution, we failed to highlight a specific effect modifier of social deprivation on SUDI in this study. However, the biological pathways underlying air pollution and social deprivation, including particularly inflammation and oxidative stress, support the main SUDI hypothesis [9, 35]. Our approach to social deprivation focused on the fdep indicator, which is very interesting but remains an ecological indicator allowing assessment of neighbourhood deprivation and does not permit direct assessment of the individual effect of social deprivation, making it difficult to draw a clear conclusion.

The multi‐pollutant analysis is consistent with the main analysis, highlighting potentially higher impacts with a lag (e.g., lag 5 or lag 3). However, while the multi‐pollutant model provides a rigorous assessment of the cumulative effect of air pollution, interpretation must remain conservative, as a simultaneous increase in all pollutants cannot be considered the norm, particularly given the negative correlation between O_3_ and NO_2_.

## 5 Conclusions

Our findings, consistent with previous studies, suggest that short‐term exposure to air pollution, particularly PM, is associated with SUDI. O_3_ should be studied more precisely across different scenarios, as it is appropriate to consider effect modifiers such as season and position. SUDI, like many multifactorial syndromes, requires a multidisciplinary approach in both research and care. As we can add other factors to these complex mechanisms, we should continue to consider both etiological and intervention strategies to limit these deaths.

## Author Contributions

**Emmanuel Bourdet:** writing – original draft, project administration, methodology, investigation, funding acquisition, formal analysis, data curation, conceptualization. **Noémie Letellier:** writing – review and editing, validation, supervision, methodology. **Tarik Benmarhnia:** writing – review and editing, validation, supervision, methodology. **Johanna Lepeule:** writing – review and editing, validation, methodology. **Bénédicte Jacquemin:** writing – review and editing, validation, methodology. **Karine Levieux:** writing – review and editing, validation, methodology. **Guillaume Barbalat:** writing – review and editing, validation, methodology. **Itai Kloog:** writing – review and editing, validation, methodology. **Rémi Béranger:** writing – review and editing, validation, methodology, investigation, funding acquisition, conceptualization. **David Boels:** writing – review and editing, validation, methodology, investigation, funding acquisition, conceptualization.

## Funding

Funds for this study were provided by a research grant from *Mécénat Santé AXA*. EB and DB received a research grant (DB) and PhD grant (EB) from *Fondation de France*.

## Conflicts of Interest

The authors declare no conflicts of interest.

## Supporting information


**Table S1:** Odds ratio and 95% confidence interval of SUDI associated with a quartile increase in mixture pollutants (Coarse fraction, PM_2.5_, NO_2_ and O_3_), adjusted for mean temperature, influenza‐like syndromes and public holidays.
**Table S2:** Odds ratio and 95% confidence interval of SUDI associated with an IQR increase in air pollutants, adjusted for mean temperature, influenza‐like syndromes and public holidays.
**Table S3:** Distribution of air pollutants by season (μg/m^3^).
**Table S4:** Results from Cochran *Q*‐test between strata.
**Figure S1:** Flowchart of data selection.
**Figure S2:** Correlations between air pollutants and with mean temperature.
**Figure S3:** Odds ratio of SUDI associated with an IQR increase in air pollutants, adjusted for mean temperature, influenza‐like syndromes and public holidays, stratified by urban or rural residence.
**Figure S4:** Odds ratio of SUDI associated with an IQR increase in air pollutants, adjusted for mean temperature, influenza‐like syndromes and public holidays, stratified by age at death.
**Figure S5:** Odds ratio of SUDI associated with an IQR increase in air pollutants, adjusted for mean temperature, influenza‐like syndromes and public holidays, stratified by biological sex.
**Figure S6:** Odds ratio of SUDI associated with an IQR increase in air pollutants, adjusted for mean temperature, influenza‐like syndromes and public holidays, stratified by sleep position at discovery.
**Figure S7:** Odds ratio of SUDI associated with an IQR increase in air pollutants, adjusted for mean temperature, influenza‐like syndromes and public holidays, stratified by social deprivation. Social deprivation was assessed using the French deprivation index (Fdep) in quintiles, with the first quintile representing the wealthiest areas and the fifth the most deprived.
**Figure S8:** Results of negative and positive weights of pollutants in the multi‐pollutant analysis.
**Figure S9:** Odds ratio of SUDI associated with an IQR increase in air pollutants, adjusted for mean temperature, influenza‐like syndromes and public holidays. Analysis includes only residence with precise GPS coordinates (*n* = 753).
**Figure S10:** Odds ratio of SUDI associated with an IQR increase in air pollutants, adjusted for mean temperature, influenza‐like syndromes and public holidays. Analysis includes all data without any exclusion (*n* = 1273).
**Figure S11:** Odds ratio of SUDI associated with an IQR increase in air pollutants, crude results without adjustments (*n* = 1078).
**Figure S12:** Results from DLM analysis on PM_10_. Overall effect includes linear dose–response, and lag‐response up to 6 days with one knot. Lag effects presented results from different exposure percentiles (50th, 75th, 90th, 95th and 99th). The placement of knots were at lag 3 for one knot, at lags 1 and 5 for two knots, and at lags 1, 3 and 5 for three knots. The overall effect was modelled using a linear dose–response, and a two knots lag‐response.
**Figure S13:** Results from DLM analysis on PM_2.5_. Overall effect includes linear dose–response, and lag‐response (3 knots) up to 6 days with one knot. Lag effects presented results from different exposure percentiles (50th, 75th, 90th, 95th and 99th). The overall effect was modelled using a linear dose–response, and a two knots lag‐response.
**Figure S14:** Results from DLM analysis on NO_2_. Overall effect includes linear dose–response, and lag‐response up to 6 days with one knot. Lag effects presented results from different exposure percentiles (50th, 75th, 90th, 95th and 99th). The placement of knots were at lag 3 for one knot, at lags 1 and 5 for two knots, and at lags 1, 3, 5 for three knots. The overall effect was modelled using a linear dose–response, and a two knots lag‐response.
**Figure S15:** Results from DLM analysis on O_3_. Overall effect includes linear dose–response, and lag‐response up to 6 days with one knot. Lag effects presented results from different exposure percentiles (50th, 75th, 90th, 95th and 99th). The placement of knots were at lag 3 for one knot, at lags 1 and 5 for two knots, and at lags 1, 3, 5 for three knots. The overall effect was modelled using a linear dose–response, and a two knots lag‐response.

## Data Availability

Data used in this study were provided by the French SUDI registry, ‘Registre OMIN’ (https://www.omin.fr/). Data are highly identifiable and are protected by both the registry's regulations and the French data protection authorities ‘CESREES/CNIL’. As such, we cannot share the data directly, but a request to access registry data can be submitted to the registry's scientific council and to ‘CESREES/CNIL’.
