# Supplementary material for: Short‐Term Effect of Air Pollutants on Sudden Unexpected Death in Infancy: A Case‐Crossover Study Based on the French National Registry
Source: Paediatr Perinat Epidemiol. 2026 Sep 7;40(8):932–40. doi: 10.1111/ppe.70196 (PMC13763138; doi:10.1111/ppe.70196)
Supplement: Supplementary file 1 [file PPE-40-932-s001.docx]

**SUPPLEMENTAL MATERIAL**

# Short-term effect of air pollutants on sudden unexpected death in infancy: A case-crossover study based on the French national registry

Emmanuel Bourdet, Noémie Letellier, Tarik Benmarhnia, Johanna Lepeule, Bénédicte Jacquemin, Karine Levieux, Guillaume Barbalat, Itai Kloog, Rémi Béranger, David Boels

**Table of contents**

**Table S1:** Odds ratio and 95% confidence interval of SUDI associated with an IQR increase in air pollutants, adjusted for mean temperature, influenza-like syndromes and public holidays.

**Table S2**: Odds ratio and 95% confidence interval of SUDI associated with an IQR increase in air pollutants, adjusted for mean temperature, influenza-like syndromes and public holidays.

**Table S3:** Distribution of air pollutants by season (µg/m^3^)

**Table S4:** Results from Cochran Q-test between strata

**Figure S1**: Flowchart of data selection

**Figure S2**: correlations between air pollutants and with mean temperature

**Figure S3:** Odds ratio of SUDI associated with an IQR increase in air pollutants, adjusted for mean temperature, influenza-like syndromes and public holidays, stratified by urban or rural residence.

**Figure S4:** Odds ratio of SUDI associated with an IQR increase in air pollutants, adjusted for mean temperature, influenza-like syndromes and public holidays, stratified by age at death.

**Figure S5:** Odds ratio of SUDI associated with an IQR increase in air pollutants, adjusted for mean temperature, influenza-like syndromes and public holidays, stratified by biological sex.

**Figure S6:** Odds ratio of SUDI associated with an IQR increase in air pollutants, adjusted for mean temperature, influenza-like syndromes and public holidays, stratified by sleep position at discovery.

**Figure S7:** Odds ratio of SUDI associated with an IQR increase in air pollutants, adjusted for mean temperature, influenza-like syndromes and public holidays, stratified by social deprivation.

**Figure S8:** Results of negative and positive weights of pollutants in the multi-pollutant analysis. The effect of each time lag was analysed separately.

**Figure S9:** Odds ratio of SUDI associated with an IQR increase in air pollutants, adjusted for mean temperature, influenza-like syndromes and public holidays. *Analysis includes only residence with precise GPS coordinates (n=753).*

**Figure S10:** Odds ratio of SUDI associated with an IQR increase in air pollutants, adjusted for mean temperature, influenza-like syndromes and public holidays. *Analysis includes all data without any exclusion (n=1,273).*

**Figure S11:** Odds ratio of SUDI associated with an IQR increase in air pollutants. *Crude results without adjustments (n=1,078).*

**Figure S12**: Results from DLM analysis on PM_10_.

**Figure S13**: Results from DLM analysis on PM_2.5_.

**Figure S14**: Results from DLM analysis on NO_2_.

**Figure S15**: Results from DLM analysis on O_3_.

**Example of R code**

**Table S1:** Odds ratio and 95% confidence interval of SUDI associated with a quartile increase in mixture pollutants (Coarse fraction, PM_2.5_, NO_2_, O_3_), adjusted for mean temperature, influenza-like syndromes and public holidays.

| Lag | OR (95% CI) |
| --- | --- |
| Lag 0 | 0.98 (0.80, 1.20) |
| Lag 1 | 1.15 (0.94, 1.40) |
| Lag 2 | 1.02 (0.84, 1.25) |
| Lag 3 | 1.22 (0.99, 1.49) |
| Lag 4 | 1.06 (0.87, 1.29) |
| Lag 5 | 1.31 (1.08, 1.60) |
| Lag 6 | 1.14 (0.94, 1.38) |
| Lag 0-1 | 1.10 (0.89, 1.35) |
| Lag 0-2 | 1.04 (0.83, 1.30) |
| Lag 0-3 | 1.03 (0.81, 1.30) |
| Lag 0-4 | 1.01 (0.79, 1.29) |
| Lag 0-5 | 1.13 (0.88, 1.15) |
| Lag 0-6 | 1.08 (0.84, 1.40) |

**Table S2**: Odds ratio and 95% confidence interval of SUDI associated with an IQR increase in air pollutants, adjusted for mean temperature, influenza-like syndromes and public holidays.

| Lag | PM_10_ | PM_2.5_ | NO_2_ | O_3_ |
| --- | --- | --- | --- | --- |
| Lag 0 | 1.05 (0.97, 1.14) | 1.04 (0.96, 1.12) | 1.12 (0.92, 1.38) | 0.93 (0.80, 1.09) |
| Lag 1 | 1.07 (0.98, 1.16) | 1.04 (0.97, 1.13) | 1.11 (0.91, 1.36) | 0.96 (0.83, 1.12) |
| Lag 2 | 1.06 (0.98, 1.16) | 1.04 (0.97, 1.12) | 1.18 (0.96, 1.45) | 1.05 (0.89, 1.22) |
| Lag 3 | 1.11 (1.02, 1.20) | 1.08 (1.00, 1.17) | 1.04 (0.85, 1.28) | 1.03 (0.88, 1.21) |
| Lag 4 | 1.11 (1.02, 1.20) | 1.09 (1.01, 1.17) | 1.05 (0.86, 1.29) | 0.98 (0.84, 1.14) |
| Lag 5 | 1.10 (1.02, 1.19) | 1.07 (1.00, 1.15) | 1.17 (0.96, 1.41) | 1.06 (0.91, 1.24) |
| Lag 6 | 1.03 (0.95, 1.12) | 1.01 (0.94, 1.09) | 1.05 (0.86, 1.28) | 1.07 (0.92, 1.25) |
| Mean lag 0_1 | 1.07 (0.98, 1.17) | 1.05 (0.97, 1.14) | 1.15 (0.91, 1.45) | 0.93 (0.79, 1.11) |
| Mean lag 0_2 | 1.08 (0.98, 1.19) | 1.06 (0.97, 1.15) | 1.22 (0.95, 1.58) | 0.97 (0.80, 1.17) |
| Mean lag 0_3 | 1.11 (1.01, 1.23) | 1.08 (0.99, 1.19) | 1.22 (0.92, 1.60) | 0.98 (0.80, 1.21) |
| Mean lag 0_4 | 1.14 (1.03, 1.28) | 1.11 (1.00, 1.22) | 1.23 (0.91, 1.65) | 0.97 (0.78, 1.21) |
| Mean lag 0_5 | 1.17 (1.05, 1.32) | 1.13 (1.02, 1.25) | 1.31 (0.96, 1.78) | 1.00 (0.79, 1.26) |
| Mean lag 0_6 | 1.18 (1.04, 1.33) | 1.12 (1.01, 1.26) | 1.31 (0.94, 1.81) | 1.02 (0.80, 1.31) |

**Table S3:** Distribution of air pollutants by season (µg/m^3^)

|  |  | Mean (SD) | Minimum | 50^th^ | Maximum |
| --- | --- | --- | --- | --- | --- |
| PM_10_ | Winter | 18.9 (10.9) | 3.2 | 16.1 | 74.6 |
|  | Spring | 16.0 (7.9) | 2.9 | 16.0 | 86.9 |
|  | Summer | 15.1 (5.8) | 4.3 | 14.1 | 49.4 |
|  | Autumn | 16.2 (7.6) | 3.6 | 14.5 | 70.3 |
| PM_2.5_ | Winter | 13.2 (9.3) | 1.8 | 10.0 | 63.6 |
|  | Spring | 9.4 (6.1) | 1.6 | 7.9 | 72.6 |
|  | Summer | 7.6 (3.5) | 2.2 | 6.8 | 27.3 |
|  | Autumn | 10.4 (6.4) | 1.7 | 8.5 | 53.0 |
| NO_2_ | Winter | 21.4 (14.2) | 0.0 | 18.8 | 81.5 |
|  | Spring | 14.9 (13.3) | 0.0 | 10.5 | 104.8 |
|  | Summer | 13.9 (12.0) | 0.0 | 10.3 | 80.4 |
|  | Autumn | 19.8 (13.6) | 0.0 | 17.4 | 68.2 |
| O_3_ | Winter | 43.8 (19.2) | 1.8 | 45.1 | 91.1 |
|  | Spring | 67.7 (15.1) | 13.1 | 67.7 | 117.1 |
|  | Summer | 64.4 (17.8) | 18.0 | 64.4 | 134.3 |
|  | Autumn | 38.5 (16.0) | 0.0 | 39.3 | 81.3 |

**Table S4**: Results from Cochran Q-test between strata

|  |  | Urban/rural | | Sleep position | | Age | | Sex | | Season | | Fdep | |
| --- | --- | --- | --- | --- | --- | --- | --- | --- | --- | --- | --- | --- | --- |
| Pollutant | Lag | Q-test result | I² | Q-test result | I² | Q-test result | I² | Q-test result | I² | Q-test result | I² | Q-test result | I² |
| PM_10_ | 0-1 | 3.8 | 73.9 | 0.2 | 0 | 0.5 | 0 | 1.2 | 13.8 | 0.9 | 0 | 3.3 | 0 |
| PM_10_ | 0-6 | 0.5 | 0 | 0.0 | 0 | 0.1 | 0 | 0.1 | 0 | 10.2* | 70.6 | 1.6 | 0 |
| PM_10_ | 3 | 0.0 | 0 | 0.4 | 0 | 0.7 | 0 | 0.7 | 0 | 4.6* | 34.4 | 2.4 | 0 |
| PM_2.5_ | 0-1 | 4.6* | 79.4 | 0.4 | 0 | 0.1 | 0 | 1.3 | 20 | 1.3 | 0 | 3.5 | 0 |
| PM_2.5_ | 0-6 | 0.8 | 0 | 0.0 | 0 | 0.0 | 0 | 0.1 | 0 | 12.8* | 76.5 | 1.5 | 0 |
| PM_2.5_ | 3 | 0.0 | 0 | 0.3 | 0 | 0.4 | 0 | 0.8 | 0 | 4.1* | 27.5 | 1.9 | 0 |
| NO_2_ | 0-1 | 1.5 | 31.5 | 1.1 | 10.7 | 0.0 | 0 | 0.4 | 0 | 0.8 | 0 | 1.0 | 0 |
| NO_2_ | 0-6 | 0.6 | 0 | 1.7 | 39.4 | 0.0 | 0 | 0.7 | 0 | 4.8* | 37.4 | 6.1* | 33.9 |
| NO_2_ | 3 | 0.9 | 0 | 4.8* | 79.3 | 0.5 | 0 | 0.2 | 0 | 5.9* | 48.7 | 4.4* | 8.5 |
| O_3_ | 0-1 | 2.3 | 56.1 | 0.0 | 0 | 0.5 | 0 | 0.4 | 0 | 6.3* | 52.5 | 5.2* | 23.7 |
| O_3_ | 0-6 | 0.5 | 0 | 0.5 | 0 | 0.5 | 0 | 1.4 | 18 | 11.2* | 73.2 | 6.2* | 35.1 |
| O_3_ | 3 | 2.2 | 55 | 2.9 | 65.4 | 0.4 | 0 | 4.2* | 75.9 | 12.8* | 76.6 | 3.8* | 0 |

Legend: Q-test result were calculated from the equation (1): [$\frac{(\beta1 - \beta p)^{2}}{VAR(\beta1)}+ \frac{(\beta2 - \beta p)^{2}}{VAR(\beta2)}+\ldots]$ adapted with the number of strata. I² were calculated using (2):$[I^{2}=\frac{\left( Q-df \right)}{Q}]$ where Q is the Q-test result.

*: p-value <0.05

**Figure S1**: Flowchart of data selection

**Figure S2**: correlations between air pollutants and with mean temperature

**Figure S3:** Odds ratio of SUDI associated with an IQR increase in air pollutants, adjusted for mean temperature, influenza-like syndromes and public holidays, stratified by urban or rural residence.

**Figure S4:** Odds ratio of SUDI associated with an IQR increase in air pollutants, adjusted for mean temperature, influenza-like syndromes and public holidays, stratified by age at death.

**Figure S5:** Odds ratio of SUDI associated with an IQR increase in air pollutants, adjusted for mean temperature, influenza-like syndromes and public holidays, stratified by biological sex.

**Figure S6:** Odds ratio of SUDI associated with an IQR increase in air pollutants, adjusted for mean temperature, influenza-like syndromes and public holidays, stratified by sleep position at discovery.

**Figure S7**: Odds ratio of SUDI associated with an IQR increase in air pollutants, adjusted for mean temperature, influenza-like syndromes and public holidays, stratified by social deprivation. *Social deprivation was assessed using the French deprivation index (Fdep) in quintiles, with the first quintile representing the wealthiest areas and the fifth the most deprived.*

**Figure S8**: Results of negative and positive weights of pollutants in the multi-pollutant analysis.

**Figure S9:** Odds ratio of SUDI associated with an IQR increase in air pollutants, adjusted for mean temperature, influenza-like syndromes and public holidays. *Analysis includes only residence with precise GPS coordinates (n=753).*

**Figure S10:** Odds ratio of SUDI associated with an IQR increase in air pollutants, adjusted for mean temperature, influenza-like syndromes and public holidays. *Analysis includes all data without any exclusion (n=1,273).*

**Figure S11:** Odds ratio of SUDI associated with an IQR increase in air pollutants, crude results without adjustments (n=1,078).

**Figure S12**: Results from DLM analysis on PM_10_. *Overall effect includes linear dose-response, and lag-response up to six days with one knot. Lag effects presented results from different exposure percentiles (50^th^, 75^th^, 90^th^, 95^th^, 99^th^). The placement of knots were at lag 3 for one knot, at lags 1 and 5 for two knots, and at lags 1, 3, 5 for three knots. The overall effect was modeled using a linear dose-response, and a two knots lag-response.*

**Figure S13**: Results from DLM analysis on PM_2.5_. *Overall effect includes linear dose-response, and lag-response (3 knots) up to six days with one knot. Lag effects presented results from different exposure percentiles (50^th^, 75^th^, 90^th^, 95^th^, 99^th^). The overall effect was modeled using a linear dose-response, and a two knots lag-response.*

**Figure S14**: Results from DLM analysis on NO_2_. *Overall effect includes linear dose-response, and lag-response up to six days with one knot. Lag effects presented results from different exposure percentiles (50^th^, 75^th^, 90^th^, 95^th^, 99^th^). The placement of knots were at lag 3 for one knot, at lags 1 and 5 for two knots, and at lags 1, 3, 5 for three knots. The overall effect was modeled using a linear dose-response, and a two knots lag-response.*

**Figure S15**: Results from DLM analysis on O_3_. *Overall effect includes linear dose-response, and lag-response up to six days with one knot. Lag effects presented results from different exposure percentiles (50^th^, 75^th^, 90^th^, 95^th^, 99^th^). The placement of knots were at lag 3 for one knot, at lags 1 and 5 for two knots, and at lags 1, 3, 5 for three knots. The overall effect was modeled using a linear dose-response, and a two knots lag-response.*

**Example of R code**

⚠ The following code snippets are provided solely for illustrative purposes and are not intended to be executed as is.

library(dplyr)
library(lubridate)
library(ggplot2)

library(survival)
library(ggpubr)

library(dlnm)

library(qgcomp)

#### **Data management**

#After matching between residence GPS coordinates and air pollution grid on QGIS, I provide an example of the database (fictive data) #WARNING: Date format is in French, so 01/05/2020 is 1st May 2020.

| ID | birth_date | death_date | model_date | pm10 |
| --- | --- | --- | --- | --- |
| 1 | 01/01/2020 | 01/05/2020 | 01/01/2020 | 10 |
| 1 | 01/01/2020 | 01/05/2020 | 02/01/2020 | 20 |
| 1 | 01/01/2020 | 01/05/2020 | 03/01/2020 | 10 |
| 1 | 01/01/2020 | 01/05/2020 | 04/01/2020 | 40 |
| … | … | … | … | … |

#So, for each ID, we observe obviously one date of birth and one date of death, and one pollutant concentration for each day available. We have to define case and control days. In the time-stratified analysis, controls are within the same day of the same month and year, and on the same day of the week as the cases.

data$year_death <- year(data$death_date)
data$month_death <- month(data$death_date)
data$wday_death <- wday(data$death_date)

data$year_model <- year(data$model_date)
data$month_model <- month(data$model_date)
data$wday_model <- wday(data$model_date)

data_cc <- data %>%
filter(year_death==year_model,
month_death==month_model,
wday_death==wday_model)

#identify case (=1) and control days (=0)

data_cc$case <- ifelse(data_cc$death_date==data_cc$model_date,1,0)

data <- data_cc

#an example of the final database (fictive data)

| ID | death_date | model_date | pm10 | case | mean_  temperature | holiday | influenza |
| --- | --- | --- | --- | --- | --- | --- | --- |
| 1 | 01/05/2020 | 01/05/2020 | 10 | 1 | 12 | 1 | 10 |
| 1 | 01/05/2020 | 08/05/2020 | 20 | 0 | 15 | 1 | 0 |
| 1 | 01/05/2020 | 15/05/2020 | 10 | 0 | 20 | 0 | 2 |
| 1 | 01/05/2020 | 22/05/2020 | 40 | 0 | 12 | 0 | 0 |
| 1 | 01/05/2020 | 29/05/2020 | 35 | 0 | 10 | 0 | 18 |
| … |  |  |  |  |  |  |  |
| 2 | 17/11/2020 | 03/11/2020 | 40 | 0 | 7 | 0 | 20 |
| 2 | 17/11/2020 | 10/11/2020 | 45 | 0 | 5 | 0 | 5 |
| 2 | 17/11/2020 | 17/11/2020 | 15 | 1 | 10 | 0 | 2 |
| 2 | 17/11/2020 | 24/11/2020 | 20 | 0 | 7 | 0 | 14 |
| … |  |  |  |  |  |  |  |

#To add the lag days, I did exactly the same with other columns (lag1_death_date, pm10_lag1, etc…)

#### **Using the basic ‘clogit’ command and adapt for all lags and pollutants**

```
model <- clogit(case~pm10 + holiday+influenza+mean_temperature+strata(ID),data=data)
summary(model)
#To assess OR and CI for an IQR increment
OR <- exp(iqr_pm10*coef(model)[1])
Lower_CI <- exp(iqr_pm10*confint(model))[1]
Upper_CI <- exp(iqr_pm10*confint(model))[4]

#This part of the code can be adapted for all lags (0,1,2,3,4,5,6) and average exposure lag (0-1,0-2,0-3,0-4,0-5,0-6). It is also possible de make a loop function
```

#### **Stratified analyses**

```
#Stratification code is provided with one example, to apply to different stratifications
#Example : strata on age
# Two category of age, from the variable “age_death” in months
polluants <- c("pm10", "pm25", "no2", "O3")
lags <- c("", "_lag0_1", "_lag1", "_lag2", "_lag3", "_lag4", "_lag5", "_lag6", "_lag0_6")
strata_var <- "age"

iqr_values <- c(
  "no2" = 17.16,
  "O3" = 28.66,
  "pm10" = 9.30,
  "pm25" = 6.83
)

results_all <- data.frame(
  Pollutant = character(),
  LagLabel = character(),
  OR = numeric(),
  Lower_CI = numeric(),
  Upper_CI = numeric(),
  Strata = character(),
  stringsAsFactors = FALSE
)

for (s in sort(unique(data[[strata_var]]))) {
  df_strata <- data %>% filter(.data[[strata_var]] == s)

  for (poll in polluants) {
    for (lag in lags) {
      tryCatch({
        var_name <- ifelse(lag == "", poll, paste0(poll, lag))
 conf_TQ <- ifelse(lag == "", "T_Q", paste0("T_Q", lag))
        conf_hol <- ifelse(lag == "", "holiday", paste0("holiday", lag))
        conf_sent <- ifelse(lag == "", "influenza", paste0("influenza", lag))

        formula_str <- paste(
          "case ~", var_name, "+",
          conf_TQ, "+",
          conf_hol, "+",
          conf_sent, "+ strata(admin_idomin.prj)")

         model <- clogit(
          formula = as.formula(formula_str),
          data = df_strata,
          method = "approximate")

         iqr_val <- iqr_values[poll]

         OR <- exp(iqr_val*coef(model)[1])
         Lower_CI <- exp(iqr_val*confint(model))[1]
         Upper_CI <- exp(iqr_val*confint(model))[4]


        lag_display <- ifelse(lag == "", "Lag 0", gsub("_lag", "Lag ", lag))

        results_all <- rbind(results_all, data.frame(
          Pollutant = poll,
          LagLabel = lag_display,
          OR = OR,
          Lower_CI = Lower_CI,
          Upper_CI = Upper_CI,
          Strata = paste0(strata_var, " = ", s),
          stringsAsFactors = FALSE))}
          , error = function(e)
          {message("Erreur : ", poll, " ", lag, " dans ", s, " - ", e$message)})}}}
```

#### **DLM analyses**

```
#I extracted a database "data_cb" with just exposure data (e.g. No2) for crossbasis

knots_lag <- c(1,3,5)
cbx <- crossbasis(data_cb, lag=maxlag,argvar=list(fun="lin"),arglag=list(fun="ns", df=, knots=knots_lag))
##for crossbasis, "lin" is for linear, and ns for splines with specific number of df (optimized with AIC). Change the number and placement of lags if df is different (for example for df=1, knots_lag <- 3)

model <- clogit(case~cbx+strata(admin_idomin.prj),data=data)
pred <- crosspred(cbx, model, bylag = 0.2, cumul = TRUE)

plot(pred, "overall", xlab="NO2", ylab ="Relative risk", main= "Overall effect",
     ylim = c(0.9, 5))

plot(pred, "slice",var=14,
     xlab = "Lag (days)",
     ylab = "RR",
     ylim=c(0.8,3),
     cumul=TRUE,
     ylab = "Relative risk",
     main = "NO2",
     col="green",ci="n")
lines(pred,"slices", var = 24, cumul = TRUE, col = "red", lwd = 2)
lines(pred,"slices", var = 36, cumul = TRUE, col = "blue", lwd = 2)
lines(pred,"slices", var = 46, cumul = TRUE, col = "purple", lwd = 2)
lines(pred,"slices", var = 60, cumul = TRUE, col = "orange", lwd = 2)
legend("topleft",
       legend = c("50th", "75th", "90th", "95th", "99th"),
       col = c("green", "red", "blue", "purple", "orange"),
       lwd = 2, cex = 0.9, bty = "n")

##different "var" here are the percentiles from No2 exposure
```

#### **Multi-pollutant analyses (quantile g computation)**

```
#Here we only use the coarse part of PM10. So create a new "coarse_pm" for each lag
data$coarse <- data$pm10-data$pm25 #To adapt for each lag

expnms <- c("coarse","pm25", "no2","O3")

fit <- qgcomp.cox.noboot(
  f = Surv(rep(1, nrow(data)), case) ~
    coarse+ no2 + O3 + mean_temperature + holiday + influenza + strata(admin_idomin.prj),
  expnms = expnms,
  data = data,
  q = 4)

summary(fit)
OR_0 <- exp(fit$coef)
low_0 <- exp(fit$ci[1])
up_0 <- exp(fit$ci[2])
fit$neg.weights
fit$pos.weights
```

**References**

1.	Kaufman JS, MacLehose RF. Which of These Things is Not Like the Others? Cancer. 2013 Dec 15;119(24):4216–22. doi:10.1002/cncr.28359 PubMed PMID: 24022386; PubMed Central PMCID: PMC4026206.

2.	Higgins JPT, Thompson SG, Deeks JJ, Altman DG. Measuring inconsistency in meta-analyses. BMJ. 2003 Sep 6;327(7414):557–60. doi:10.1136/bmj.327.7414.557 PubMed PMID: 12958120; PubMed Central PMCID: PMC192859.
